# Decyl caffeic acid inhibits the proliferation of colorectal cancer cells in an autophagy-dependent manner *in vitro* and *in vivo*

**DOI:** 10.1371/journal.pone.0232832

**Published:** 2020-05-13

**Authors:** Ching Chen, Yueh-Hsiung Kuo, Cheng-Chieh Lin, Che-Yi Chao, Man-Hui Pai, En-Pei Isabel Chiang, Feng-Yao Tang

**Affiliations:** 1 Biomedical Science Laboratory, Department of Nutrition, China Medical University, Taichung, Taiwan, Republic of China; 2 Department of Chinese Pharmaceutical Sciences and Chinese Medicine Resources, China Medical University, Taichung, Republic of China; 3 Department of Biotechnology, Asia University, Taichung, Taiwan, Republic of China; 4 School of Medicine, College of Medicine, China Medical University, Taichung, Taiwan, Republic of China; 5 Department of Family Medicine, China Medical University Hospital, Taichung, Taiwan, Republic of China; 6 Department of Healthcare Administration, College of Health Science, Asia University, Taichung, Taiwan, Republic of China; 7 Department of Food Nutrition and Health Biotechnology, Asia University, Taichung, Taiwan, Republic of China; 8 Department of Medical Research, China Medical University Hospital, China Medical University, Taichung, Taiwan, Republic of China; 9 Department of Anatomy and Cell Biology, School of Medicine, College of Medicine, Taipei Medical University, Taipei, Taiwan, Republic of China; 10 Department of Food Science and Biotechnology, National Chung Hsing University, Taichung, Taiwan, Republic of China; 11 Innovation and Development Center of Sustainable Agriculture (IDCSA), National Chung Hsing University, Taichung, Taiwan, Republic of China; Columbia University, UNITED STATES

## Abstract

The treatment of human colorectal cancer (CRC) cells through suppressing the abnormal survival signaling pathways has recently become a significant area of focus. In this study, our results demonstrated that decyl caffeic acid (DC), one of the novel caffeic acid derivatives, remarkedly suppressed the growth of CRC cells both *in vitro* and *in vivo*. The inhibitory effects of DC on CRC cells were investigated in an *in vitro* cell model and *in vivo* using a xenograft mouse model. CRC cells were treated with DC at various dosages (0, 10, 20 and 40 μM), and cell survival, the apoptotic index and the autophagy level were measured using an MTT assay and flow cytometry analysis, respectively. The signaling cascades in CRC were examined by Western blot assay. The anti-cancer effects of DC on tumor growth were examined by using CRC HCT-116 cells implanted in an animal model. Our results indicated that DC differentially suppressed the growth of CRC HT-29 and HCT-116 cells through an enhancement of cell-cycle arrest at the S phase. DC inhibited the expression of cell-cycle regulators, which include cyclin E and cyclin A proteins. The molecular mechanisms of action were correlated to the blockade of the STAT3 and Akt signaling cascades. Strikingly, a high dosage of DC prompted a self-protection action through inducing cell-dependent autophagy in HCT-116 cells. Suppression of autophagy induced cell death in the treatment of DC in HCT-116 cells. DC seemed to inhibit cell proliferation of CRC differentially, and the therapeutic advantage appeared to be autophagy dependent. Moreover, consumption of DC blocked the tumor growth of colorectal adenocarcinoma in an experimental animal model. In conclusion, our results suggested that DC could act as a therapeutic agent through the significant suppression of tumor growth of human CRC cells.

## Introduction

Many studies demonstrate that colorectal cancer (CRC) is one of the most common cancer types with a high mortality rate globally [1]. Traditional chemotherapy is still the preferred treatment for CRC. However, it is well known that features of chemotherapy include low selectivity and systemic toxicity [2]. Moreover, this therapeutic remedy has many nasty side effects [2]. Due to the limitations and drawbacks of chemotherapy, the development of molecular targeted agents remains in demand.

During tumor development, abnormal triggering of the phosphatidylinositol -3-kinase (PI3-K), Akt, the mammalian target of rapamycin (mTOR) and the STAT3 survival pathways is usually observed in many cancer cell types [3]. Several studies suggested that the Akt, mTOR and STAT3 cascades contributed to cell proliferation and to the high resistance to cellular apoptosis in CRC cells [4, 5]. The Akt/mTOR signaling pathway is a considerable regulator for the biosynthesis of protein [6] and plays an important role in controlling cell growth in various types of cancer cells [7]. Activation of the Akt/mTOR pathway is often correlated with tumor growth [8], while the suppression of Akt shows promising tools for cancer cell treatment [9]. Recent studies indicated that the STAT3 signaling pathways are also considered as crucial targets for CRC treatment [10]. Thus, exploring novel antagonists of the Akt, mTOR and STAT3 cascades should be helpful in pursuing drug development and the cure of CRC. Previous studies have showed that the cell cycle progression at the S phase is mainly modulated by the cellular levels of cyclin A proteins [11]. It is already known that the excessive expression of the cyclin A protein enhances cancer progression. The downregulation of cyclin A protein would block cell cycle progression and cause an cell cycle arrest at the S phase [12, 13].

Previous studies demonstrated that the PI3-K/Akt signaling pathway is associated with the autophagy process [14]. Studies suggested that autophagy affects cell survival through the clearance of defective organelles and the preservation of cell bioenergetics in human cells [15]. During the autophagy process, Beclin-1 and LC3A/B play important roles in the catabolic pathway for cell degradation of defective organelles and macromolecules [16, 17]. A recent study indicated that an acquired-resistance to anti-EGFR therapy is associated with an increasing level of autophagy in several types of cancer [18, 19]. Silencing key autophagy proteins such as Beclin-1 would further induce cell apoptosis in CRC cells [14].

**Fig 1:**
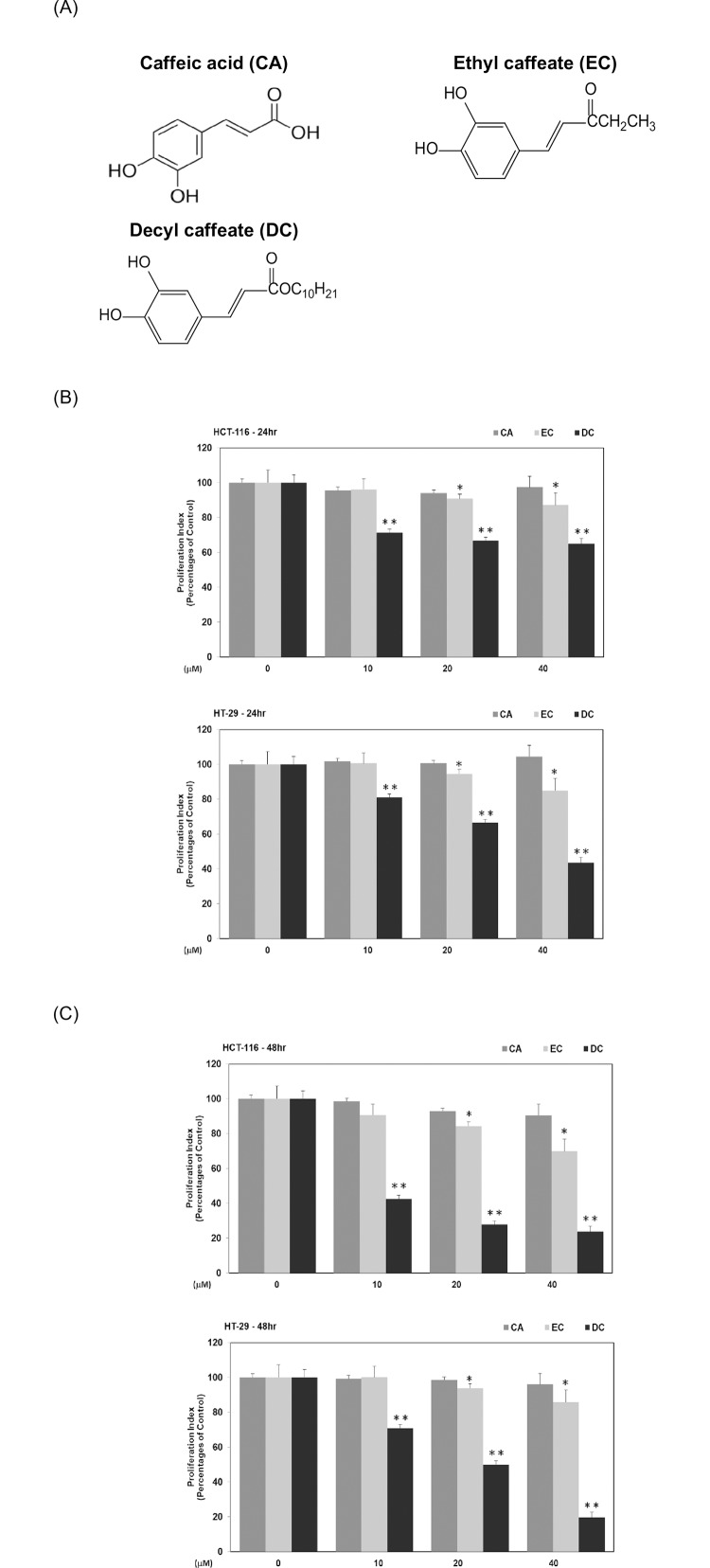
Inhibitory effects of caffeic acid derivatives on the proliferation of CRC cells *in vitro*. (A) The structures of CA, EC and DC. Human CRC cells such as HCT-116 or HT-29 cells were cultured in McCoy’s 5A medium with CA, EC or DC (at concentrations of 0, 10, 20 and 40 μM) for 24 hr (B) and 48h (C). MTT assay was performed to measure cell survival as described in Materials and Methods. Statistical analysis is expressed as the mean ± SD (standard deviation) of three independent experiments. In EC treatment subgroup, a single asterisk (*) represents a statistically significant difference in comparison with the control subgroup (*P*<0.05). In DC treatment subgroup, double asterisks (**) indicate a statistically significant difference in comparison with the control subgroup (*P*<0.05).

Previously, our results showed that caffeic acid phenethyl ester (CAPE), a well-known derivative of CA, effectively inhibited the survival of human CRC cells [20]. Ethyl caffeic acid (EC) and decyl caffeic acid (DC) (Fig 1A) are derivatives of CA for which mechanisms to inhibit the growth of CRC cells have not yet been demonstrated. In order to determine whether the CA derivative may have the therapeutic potential to prevent the growth of CRC *in vivo*, animal studies were undertaken, with major findings demonstrated in this study. Herein, we examine the molecular mechanisms of action by which EC and DC suppress the growth of CRC cells *in vitro* and *in vivo*.

We adopted CRC HCT-116 (*KRAS* gene mutant) and HT-29 (*KRAS* gene wild-type) cells as our major cell models. Our results indicated that EC and DC inhibited the proliferation of CRC cells in a dose-dependent manner *in vitro*. Among them all, DC was determined to be effective against HCT-116 and HT-29 cells at an IC_50_ of 14 μM and 10 μM, respectively. Briefly, the suppressive effects of DC on cell survival was through cell-cycle arrest at the S phase and inactivation of Akt and STAT3 proteins in CRC cells. Strikingly, a high dosage of DC carried out the formation of autophagy in HCT-116 cells rather than in HT-29 cells. The blockade of autophagy increased sensitivity to DC and further caused cell apoptosis in HCT-116 cells. Furthermore, DC inhibited the growth of colorectal carcinoma in experimental animals that were transplanted with CRC HCT-116 cells. These *in vitro* and *in vivo* findings confirm that DC noticeably inhibits tumor growth and has potential as a therapeutic agent to kill human CRC cells.

## Materials and methods

### Materials, cell lines and reagents

Authenticated human CRC cell lines such as HCT-116 cells (CCL-247) featured with *KRAS* gene mutant and HT-29 cells (HTB-38) featured with *KRAS* gene wild-type were acquired from American Type Culture Collection (Walkersville, MD). The following antibodies were obtained from Santa Cruz Biotechnology, Inc. (Dallas, TX): anti-phosphorylation-Akt Ser473 (p-Akt S473), anti-total-Akt (Akt), anti- phosphorylation -STAT3 Ser9(p-STAT3 S9), anti-total-STAT3 (STAT3), anti-cyclin A, anti-Atg3, anti-Atg5, anti-Atg12, anti-Atg16, anti-Beclin-1, anti-LC3A/B, anti-cyclin B, anti-cyclin E, anti-cdk2, and anti-Lamin A monoclonal antibodies. Acridine Orange (AO), 3-methyladenine (3-MA), caffeic acid (CA), anti-actin antibody, dimethyl sulfoxide (DMSO), hematoxylin solution and eosin solution (H&E) were purchased from Sigma-Aldrich Inc. (St Louis, MO). The reagent kit for the extraction of nuclear and cytoplasic proteins was purchased from Pierce Biotechnology Inc. (Lackford, IL). Propidium Iodide (PI) and matrigels^®^ was purchased from BD Biosciences Inc. (Franklin Lakes, NJ). McCoy’s 5A medium and fetal bovine serum (FBS) were purchased from Invitrogen Inc. (Carlsbad, CA). EC and DC (Fig 1A) were acquired from Dr. Y. H. Kuo (China Medical University, Taichung, Taiwan), one of the co-authors of this research work.

### Cell culture and treatment of CA derivatives

Human CRC cells were cultured and maintained in McCoy’s 5A medium provided with 10% heat-inactivated FBS, L-glutamine (2 mM) and sodium bicarbonate (1.5 g/L). CRC cells were treated with various dosages (0, 10, 20 and 40 μM) of CA or CA derivatives. For efficient treatment of CRC cells, CA and CA derivatives were dissolved in DMSO and mixed with FBS and culture medium. In the control subgroup, an equivalent volume of solvent (DMSO) was provided as a carrier vehicle (final concentration: 0.05% v/v).

### Analysis of cell proliferation

In this study, we performed the MTT (3-[4,5-dimethhylthiaoly]- 2,5-diphenyltetrazolium bromide) assay to measure the cell proliferation level. Human CRC cells including HCT-116 or HT-29 cells (1x 10^5^ cells / well) were sub-cultured into a 24-well plate and grown in media containing CA, EC or DC (at concentrations of 0, 10, 20 and 40 μM). To study the role of Akt in DC-treated CRC cells, CRC cells were treated with DC (40μM) in the presence or absence of wortmannin (20 μM). To confirm the effects of autophagy in cell proliferation, CRC cells were treated with DC (0 and 40μM) in the presence or absence of 3-MA (0, 1, 2, and 5 mM). To investigate whether overexpression of Akt protein could modulate cell viability in DC-treated cells, CRC cells were transfected with Active-Akt plasmid (obtained from Addgene, Cambridge, MA) by using Neon transfection system (ThermoFisher Scientific, Waltham, MA) and measured the cell survival rate. The cell proliferation assay was performed in a triplicate test. After 24 hr and 48 hr, culture media were replaced with 0.5 mg/mL MTT working solution in each well. At the end of incubation, the MTT working solution was discarded and replaced with isopropanol in each well. These 24-well plates were shaken on a vibrating device to dissolve the precipitation crystals. Optical density was measured at wavelength of 570 nm with a multi-channel filter plate reader.

### Analysis of cell-cycle progression

Human CRC cells (1x10^6^ cells/plate) were cultured in 3-cm culture plates. Cells were cultured in 0.5% FBS McCoy’s 5A medium to synchronize to the same cell-cycle stage before the experiment. To measure the effects of EC and DC on the cell-cycle distribution, human CRC cells were incubated with EC or DC (0, 10, 20 and 40 μM) in 10% FBS McCoy’s 5A medium for 24hr. At the end of the experiment, CRC cells collected from each plate by using a trypsin/EDTA solution were mixed with the binding buffer, stained with PI solution in the dark and analyzed using a FACSCanto flow cytometry system (BD Biosciences Inc., Franklin Lakes, NJ). The measurement and analysis of the PI-staining in human CRC cells was performed using the manufacturer’s software (FACSCanto flow cytometry software).

### Detection of cellular autophagy by Acridine Orange staining

Acridine Orange (AO) staining was performed as described in a previous protocol [21]. Briefly, human CRC cells were treated with DC (at dosages of 0, 10, 20 and 40 μM) in 10% FBS McCoy’s 5A medium for 24 hr. To further examine the role of autophagy, human CRC cells were treated with DC (at dosages of 0 and 40 μM) in the presence or absence of 1 mM 3-MA for 24 hr. At the end of the treatment, the cells were collected by trypsinization, stained with AO (1 μg/mL) in staining solution at room temperature and analyzed using a FACSCanto flow cytometry system. Data were analyzed using InCyte 2.6 Software (Incyte Inc. Wilmington, DE).

### Detection of cellular apoptosis by Annexin-V-propidium iodide-binding assay

The apoptotic levels of HCT-116 cells were measured by using a commercial detection kit (Invitrogen, Carlsbad, CA). After incubation with DC (0 and 40 μM) and different concentrations of 3-MA (0, 1 and 2 mM), the suspended HCT-116 cells (5X10^6^ cells/plate) were collected and mixed with binding buffer in each Eppendorf tube. FITC-Annexin V and propidium iodide (PI) working solutions were mixed with cells in each Eppendorf tube. After mixing with these reagents, cells were analyzed for cell cycle distribution using flow cytometry. The Annexin-V binding levels were measured by an FITC signal detector. The PI staining levels were measured by a phycoerythrin emission signal detector.

### A xenograft model for tumor growth

Four-week old female nude mice (body weight 17–22 g) were obtained from the National Animal Center in Taipei (Taiwan). This research is accorded with internationally accepted principles for animal use and care, as reviewed and approved by the institutional Animal Care and Use Committee Guidelines of China Medical University (IACUC, CMU) (animal protocol number: 104-153-N). These nude mice were raised in an institutional facility under specific pathogen-free (SPF) regulations. A Lab 5010 diet (from LabDiet Inc., St. Louis, MO, USA) was given to mice during the entire experimental period. All mice were anesthetized by inhalation of isoflurane during the experimental procedure according to animal protocol regulations.

Human CRC cells were detached from the culture flask and collected by treatment with trypsin/EDTA solution. Trypsinization of CRC cell suspensions was terminated with FBS containing medium. HCT-116 cells (1 x 10^6^ cells/0.1 ml medium) were inoculated into the right flank of nude mice to establish a mouse xenograft model. After the inoculation of HCT-116 cells, these nude mice (a total of 18 mice) were randomly divided into three subgroups (n = 6 in each subgroup). DC was given to those nude mice in the experimental subgroups by daily gavage. Low level (Low-DC) and high level (High-DC) of DC subgroups received a daily feeding of DC at dosages of 0.2 mg /kg of body weight (BW) and 2mg/kg of BW in corn oil, respectively. The tumor (control) subgroup only received equal amount of corn oil in the absence of DC. To avoid confounding factors, BW and amount of food intake were measured every week in those mice. No significant differences in BW or food intake among these subgroups were observed in this study.

### Hematoxylin-eosin staining of tumor tissues

For histopathological staining, frozen tumor tissues and hepatic tissues were sectioned, fixed with paraformaldehyde solution (at the concentration of 4%) and stained with H&E solution for the assessment of tumor malignancy and toxicity. Images from six randomly selected fields were recorded in a single-blinded procedure in each tissue section under an Olympus BX-51 microscope at a power field of 200X (Olympus, Tokyo, Japan). Duplicate slices of each of six different tissue samples from the control and experimental subgroups were documented and analyzed using an Olympus imaging system (Olympus, Tokyo, Japan).

### Protein preparation and Western blotting analysis

Cytoplasmic and nuclear proteins of human CRC cells were prepared using the commercial extraction kit (NE-PER^™^ Kit) with protease and phosphatase inhibitors. During the protein preparation, cell lysates were centrifuged at 12,000 x g for 10 min to separate the cytoplasmic protein from the nuclear one. After the centrifugation, the supernatant was collected as a cytoplasmic extract. The remaining precipitation was dissolved in lysis buffer as a nuclear extract for further analysis.

Cytoplasmic proteins (60 μg) fractionated by using10% SDS-PAGE were transferred to poly-vinylidene fluoride (PVDF) blotting membranes and detected by probing with specific monoclonal antibody against phosphorylation-Akt (anti-p-Akt S473). The remaining proteins in the cell lysates were measured using antibodies of anti-t-Akt, anti-p-STAT3 (Ser9), anti-t-STAT3, anti-Atg3, anti-Atg5, anti-Atg12, anti-Atg16, anti-Beclin-1, anti-LC3 A/B, anti-cyclin A, anti-cyclin E, anti-cyclin B and anti-CDK2. These blotting membranes were stripped with washing solution and re-probed with internal cytoplasmic and nuclear control antibodies against actin and lamin A proteins, respectively.

### Analysis of biostatistics

A biostatistical analysis was performed to determine the significant difference of the cell viability in CRC cells between control and experimental subgroups using SYSTAT software. We used one-way ANOVA model to confirm a significant difference which required an exclusion of null difference between the mean values originated from different subgroups at the *P* = 0.05 level. A Duncan’s multiple range test was applied to evaluate differences among these subgroups.

## Results

### Inhibitory effects of caffeic acid derivatives on the proliferation of CRC cells *in vitro*

In this study, we first investigated the inhibitory effects of CA and its derivatives, including Ethyl caffeic acid (EC) and Decyl caffeic acid (DC) (Fig 1A) on the proliferation of human CRC cells (HT-29 and HCT-116) *in vitro*. As shown in Fig 1B and 1C, EC and DC (at the concentrations of 10, 20 and 40 μM) significantly inhibited the proliferation of human CRC HT-29 and HCT-116 cells at different time points (*P*<0.05). DC was more effective than EC in suppressing cell proliferation in human CRC cells. In the case of human CRC HCT-116 cells, DC (at the concentrations of 10, 20 and 40 μM) inhibited cell proliferation up to 29%, 33.3% and 35% at the 24hr time point, respectively (Fig 1B). DC further inhibited cell proliferation up to 58%, 73% and 77% at the 48 hr time point, respectively (Fig 1C). For human HT-29 cells, DC at the same concentrations suppressed cell proliferation up to 20%, 34% and 57% at the 24hr time point, respectively (Fig 1B). DC further blocked cell proliferation up to 30%, 50% and 81% at the 48 hr time point, respectively (Fig 1C).

The respective IC_50_ values of CA, EC and DC for human CRC HCT-116 cells was 116, 90 and 14 μM. The respective IC_50_ values of CA, EC and DC for human CRC HT-29 cells were 101, 74 and 10 μM. The current study demonstrates that DC is more effective than CA and EC on the suppression of cell proliferation in human CRC cells.

### DC induces cell-cycle arrest at the S phase through the suppression of cyclin A protein in CRC cells

**Fig 2:**
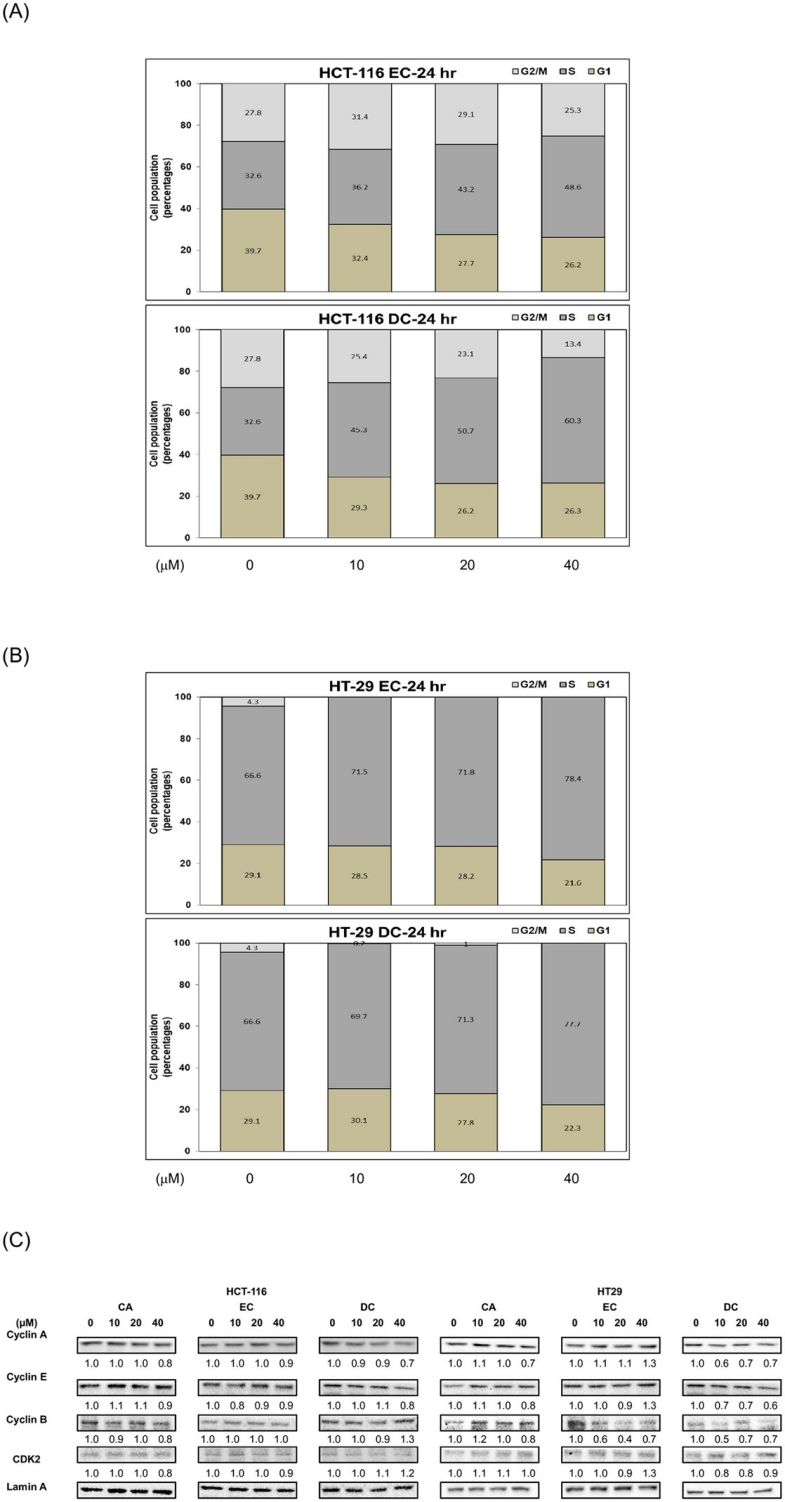
DC induces cell-cycle arrest at the S phase through the suppression of cyclin A protein in CRC cells. For the measurement of cell cycle analysis, human CRC cells were cultured in the presence or absence of EC or DC (0, 10, 20 and 40 μM) and cultured in 10% FBS McCoy’s 5A medium for 24 hr. The analysis of the distribution of different cell cycle phases was performed using flow cytometry, as described in Materials and Methods in HCT-116 cells (A) or HT-29 (B) cells. The quantitative results of cell cycle analysis were demonstrated. (C) Human CRC cells were cultured in 10% FBS McCoy’s 5A medium and treated with CA, EC or DC (0, 10, 20 and 40 μM) for 24 hr. Nuclear proteins were collected for Western blotting analysis using monoclonal antibodies against cyclin A, cyclin E, cyclin B, CDK2 and lamin A as described in Materials and Methods. The levels of detection represented the amount of cyclin A, cyclin E, cyclin B and CDK2 in the nuclei of human CRC HCT-116 and HT-29 cells.

We further investigated the distribution of the cell cycle in CRC cells to study the mechanisms underlying the inhibitory effects of DC on cell proliferation. After the treatment of DC, CRC cells stained with PI were measured for the distribution pattern of the cell cycle using flow cytometry at the 24hr time point (Fig 2A and 2B). With respect to human HCT-116 cells, DC (at the concentrations of 10, 20 and 40 μM) significantly induced cell-cycle arrest at the S phase by up to 45.3%, 50.7% and 60.3%, respectively (*P*<0.05). However, only 32.6% of the cells in the control group were in the S phase (Fig 2A). Moreover, DC also induced cell-cycle arrest in HT-29 cells in the S phase (Fig 2B). DC (at the concentrations of 10, 20 and 40 μM) significantly induced cell-cycle arrest at the S phase by up to 69.7%, 71.3% and 77.7% in comparison with 66.6% of cells in the control group, respectively (Fig 2B). Our results indicated that DC mainly suppressed the cell proliferation of CRC HCT-116 and HT-29 through the S phase cell-cycle arrest.

To further determine the mechanisms of action, we examined the inhibitory effects of DC on the expression of cell-cycle-related proteins in CRC cells. As shown in Fig 2C, DC significantly reduced the expression of the cyclin A and cyclin E proteins in both HCT-116 and HT-29 cells at the 24hr time point. These results indicate that DC effectively induces cell-cycle arrest through a reduction of the nuclear cyclin A and cyclin E protein levels in both HCT-116 and HT-29 cells.

### DC inhibited the cell proliferation of CRC cells through the inactivation of Akt protein

**Fig 3:**
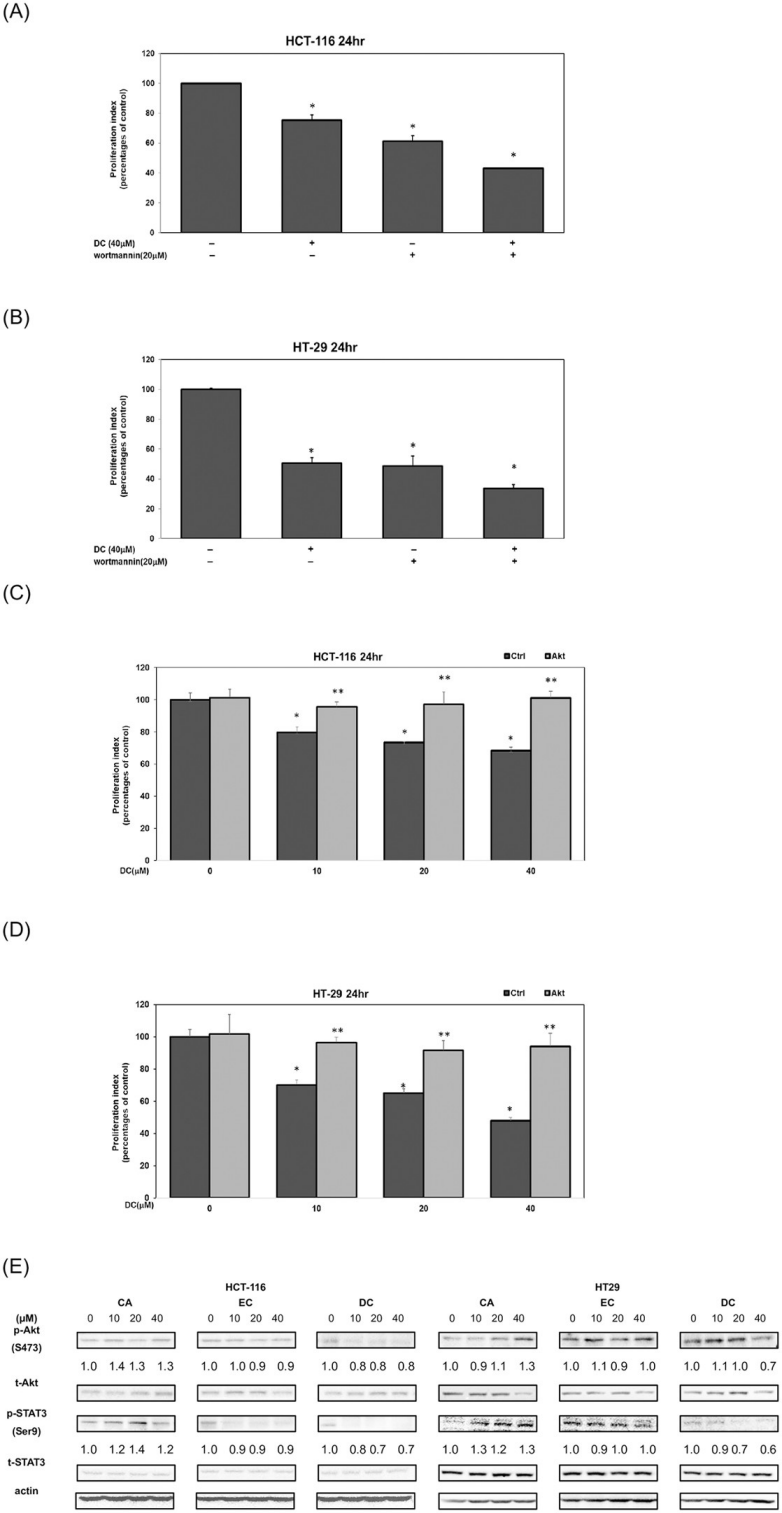
DC inhibited the cell proliferation of CRC cells through the inactivation of Akt protein. Human CRC HCT-116 (A) and HT-29 (B) cells treated with either DC (40 μM) or wortmannin (20 μM) and in 10% FBS McCoy’s 5A medium for 24hr. The cell proliferation was measured by MTT assay as described in Materials and Methods. Transfection of HCT-116 (C) and HT-29 (D) cells with active Akt plasmids was performed as described in Materials and Methods. The effect of overexpressed active-Akt plasmid in the treatment of DC (0, 10, 20 and 40 μM) in CRC cells was analyzed by using MTT assay. Statistical analysis is expressed as the mean ± SD (standard deviation) of two independent experiments. A single asterisk (*) indicates a significant difference of DC-treatment group in comparison to the untreated control subgroup in human CRC cells. Double asterisks (**) indicate a significant difference of Akt overexpression in comparison to the DC-treated human CRC cells at the same concentration subgroup. (E) Western blot analysis of cytoplasmic proteins (at 1 hr time point) using monoclonal antibodies against p-Akt (Ser473), p-STAT3 (Ser9) and corresponding controls (t-Akt, t-STAT3). Band intensities represent the amounts of p-Akt (Ser 473) and p-STAT3 (Ser9) in the cytoplasm of CRC cells.

Previous studies reported that the activation of the PI3-K/Akt signaling cascades plays a critical role in the regulation of cell survival in human CRC cells. To study the mechanism of actions, we further investigated the effects of DC on the cell survival signaling pathways in human CRC cells. As shown in Fig 3, treatment with DC or wortmannin (a specific Akt inhibitor) significantly suppressed the cell survival of HCT-116 cells (Fig 3A) and HT-29 cells (Fig 3B) at the 24hr time point. To further confirm the mechanism of action, an MTT assay was performed on HCT-116 cells following the transfection with an active Akt plasmid. As shown in Fig 3, the transfection of active Akt plasmid restored cell survival in DC-treated HCT-116 cells (Fig 3C) and HT-29 cells (Fig 3D). These results suggested that DC may inhibit the proliferation of HCT-116 cells in part through the suppression of the Akt signaling cascade.

Therefore, we further examined the inhibitory effects of DC on these cell-survival signaling pathways in CRC HCT-116 and HT-29 cells. As shown in Fig 3E, the treatment of DC inhibited the phosphorylation of Akt protein in comparison with the untreated control group in CRC cells (Fig 3E). Interestingly, DC inhibited the phosphorylation of STAT3 protein in HCT-116 and HT-29 cells. These results indicate that DC-mediated suppression of cell survival in CRC cells was associated with the inactivation of the Akt signaling protein.

### Autophagy plays an important role in DC-mediated cell cytotoxicity in HCT-116 cells

**Fig 4:**
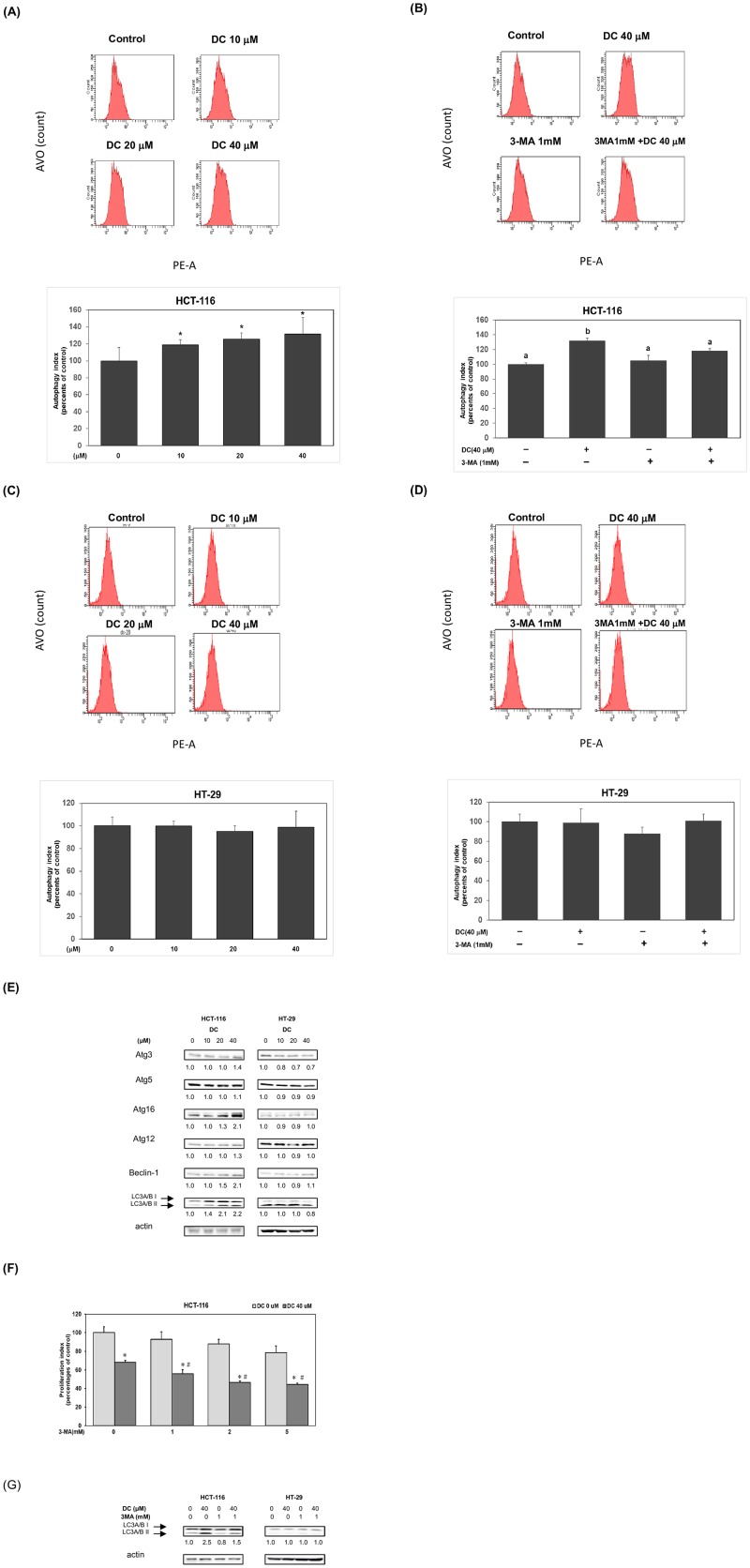
Autophagy plays an important role in DC-mediated cell cytotoxicity in HCT-116 cells. HCT-116 cells (A) and HT-29 cells (C) were treated with DC (0, 10, 20 and 40 μM) for 24hr and stained with AO for analysis of autophagy by using flow cytometry. The analysis of autophagy formation was performed as described in Materials and Methods section. A single asterisk (*) indicates a significant difference of autophagy levels in comparison to the control group in HCT-116 cells. HCT-116 cells (B) or HT-29 cells (D) were treated with 40 μM of DC in the presence or absence of 3-MA (1mM) by using similar procedure. Different letters indicate a significant difference of autophagy levels among different subgroups. (E) Western blot analysis of cytoplasmic proteins (at 24hr time point) using monoclonal antibodies against Atg3, Atg5, Atg16, Atg12, Beclin-1, LC3A/B and corresponding internal control actin protein. Band intensities represent the amounts of Atg3, Atg5, Atg16, Atg12, Beclin-1 and LC3A/B proteins in the cytoplasm of CRC cells. (F) Human CRC HCT-116 cells were incubated with 40 μM of DC in the presence or absence of 3-MA (0, 1, 2 and 5 mM) for 24 hr time points. The effect of 3-MA was measured by using MTT assay described above. Statistical analysis is expressed as the mean ± SD (standard deviation) of two independent experiments. A single asterisks (*) indicates a within-subgroup significant difference between DC (40 μM) and DC (0 μM) subgroup at each 3-MA subgroup in HCT-116 cells. A pound sign (#) indicates an inter-subgroup significant difference among 3-MA treatment in the presence of DC (40 μM) in HCT-116 cells. (G) Western blot analysis of cytoplasmic proteins (at 24hr time point) using monoclonal antibodies against LC3A/B and corresponding internal control actin protein.

Our results suggest differential effects of DC on the suppression of cell survival in HCT-116 and HT-29 cells. Previous studies indicated that autophagy plays an important role in cell survival [14]. To verify the probable mechanisms, we further investigated whether DC could induce cell autophagy in different CRC cells. As shown in Fig 4A, DC significantly induced autophagy in HCT-116 cells in a dose-dependent manner. Treatment of 3-MA, an inhibitor of autophagy, significantly inhibited autophagy formation in DC-treated HCT-116 cells (Fig 4B). Interestingly, a similar procedure of DC treatment didn’t induce cell autophagy in HT-29 cells (Fig 4C). Neither treatment of 3-MA could further affect autophagy formation in the presence or absence of DC in HT-29 cells (Fig 4D).

To verify our findings, we further examined the effects of DC on the expression of autophagy proteins. As shown in Fig 4E, DC induced the expression of autophagy proteins including Atg3, Atg16, Beclin-1 and LC3A/B in HCT-116 cells but not in HT-29 cells. These results demonstrated that DC induced cell autophagy in part through an increased expression of autophagy proteins (Atg3, Atg16, Beclin-1 and LC3A/B) in HCT-116 cells. Therefore, we further investigated whether autophagy played an important role in determining cell viability by using an autophagy antagonist. As shown in Fig 4F, treatment of 3-MA (1, 2 and 5 mM) reduced the cell viability in comparison with control subgroup (3-MA 0 mM; DC 40 μM). As shown in Fig 4G, treatment with 3-MA further inhibited the expression of LC3A/B proteins in HCT-116 cells. These results suggested that DC-mediated cell autophagy allowed them to obtain an acquired resistance phenotype in HCT-116 cells. The blocking of autophagy would further enhance cell death under DC treatment in HCT-116 cells.

### Suppression of cell autophagy induced apoptosis in DC-treated HCT-116 cells

**Fig 5:**
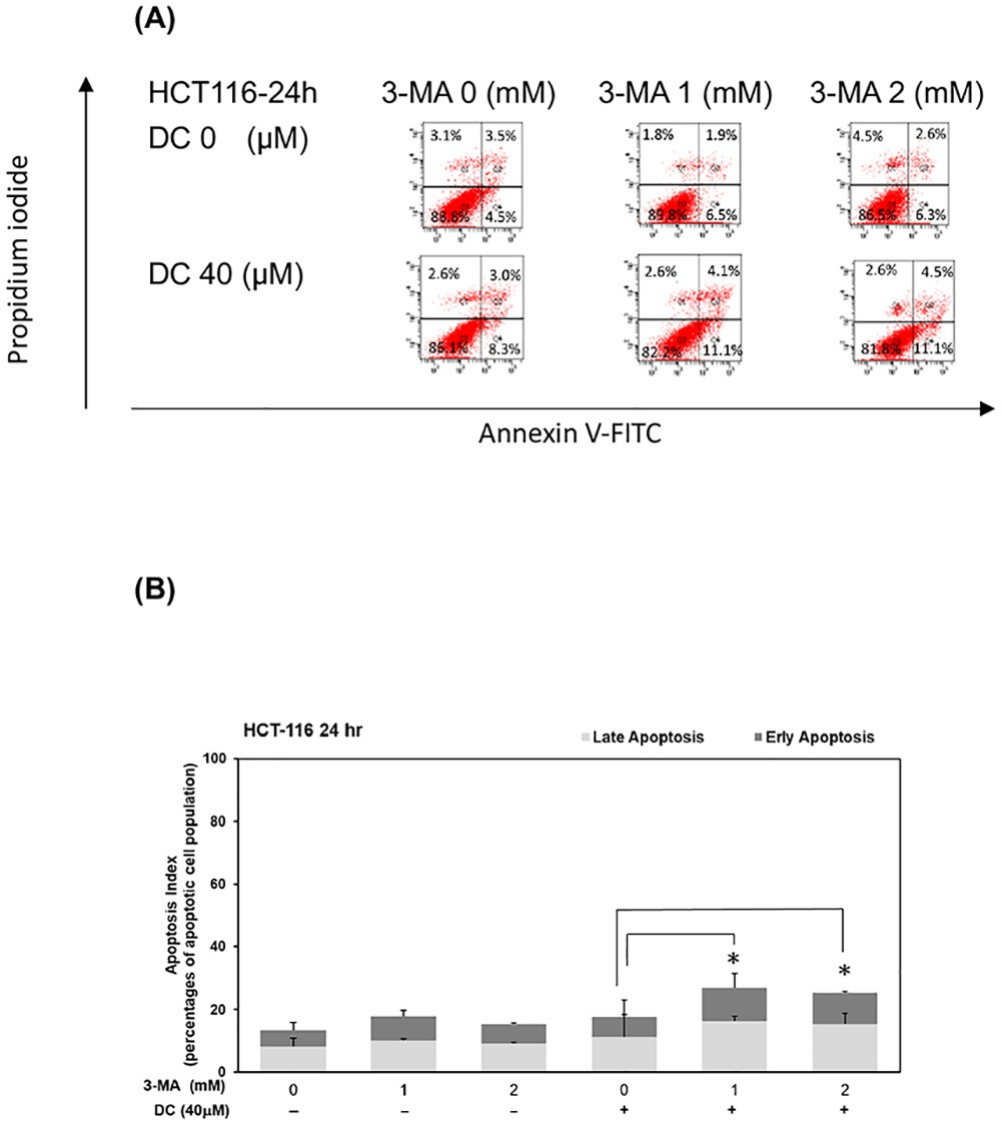
Suppression of cell autophagy induced apoptosis in DC-treated HCT-116 cells. (A) HCT-116 cells treated with DC (at concentrations of 0 and 40 μM) in the presence of 3-MA (at concentrations of 0, 1 and 2 mM) for 24hr. To determine whether 3-MA affected the apoptosis of HCT-116 cells, the apoptotic rate of HCT-116 cells was detected by the Annexin V-FITC Apoptosis Detection Kit according to the manufacturer’s instruction. The quantitative results at 24 hr (B) time points were described as percentage of apoptotic cell population. A single asterisks (*) indicates a significant difference of apoptotic levels in comparison to the DC-treated only subgroup.

We further investigated whether autophagy plays an important role in determining cell death in DC-treated HCT-116 cells. As shown in Fig 5A, treatment with 3-MA significantly augmented apoptosis in the presence of DC (40 μM) in HCT-116 cells (*P*<0.05). Treatment with 3-MA significantly induced apoptosis in the presence of DC (40 μM) in HCT-116 cells at 24hr (Fig 5B) (*P*<0.05). These results suggested that the suppression of autophagy would induce cell death through an induction of apoptosis under DC treatment in HCT-116 cells.

### Consumption of DC inhibits the growth of colorectal carcinoma in a mouse xenograft model

**Fig 6:**
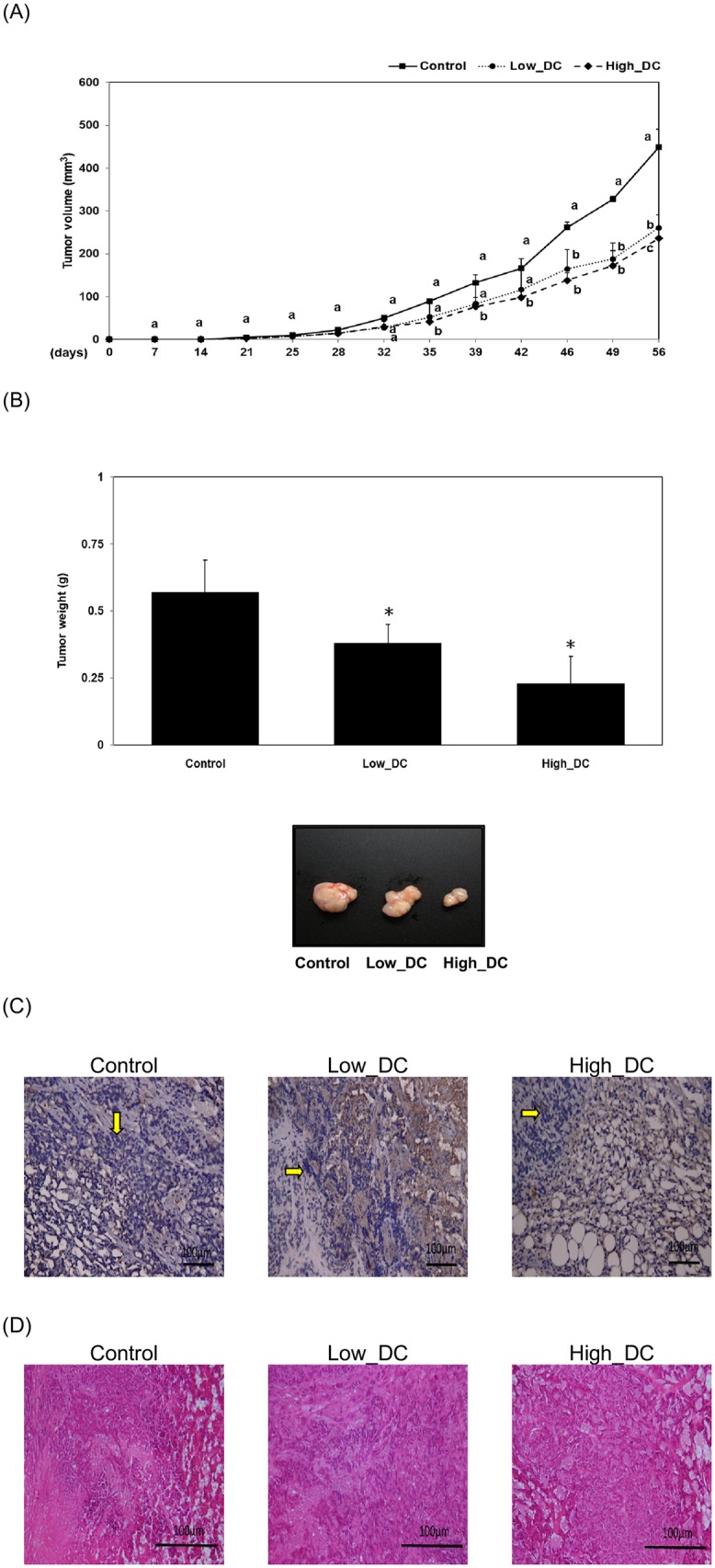
Consumption of DC inhibits the growth of colorectal carcinoma in a mouse xenograft model. Xenograft nude mice (n = 6 for each subgroup) were divided into three subgroups (the tumor subgroup, Low-DC, High-DC) and given DC (at a dosage of 0, 0.2 and 2 mg /kg of body weight (BW) per day) for 8 weeks. Data represent the change in the tumor volume (A) and tumor weight (B) among the tumor subgroup (i.e. the control subgroup), tumor with Low-DC dosage (0.2 mg /kg of BW per day) and tumor with High-DC dosage (2 mg /kg of BW per day). For the analysis of tumor volume, different letters represent a significant difference among different subgroups at the same time points (P*<0*.*05)* (A). For the analysis of tumor weight, a single asterisk (*) indicates a significant difference in comparison to the control subgroup (P*<0*.*05*) (B). (C) Tumor tissues or (D) hepatic tissues were fixed, sectioned and stained as described in the Materials and Methods. Blue spots (indicated with yellow arrows) represent the nuclei stained with hematoxylin (C). (D) No difference was found among different subgroups.

To confirm these *in vitro* findings, we further examined the inhibitory effects of DC on the growth of HCT-116 cells in a mouse xenograft model. As shown in Fig 6A, consumption of DC inhibited the growth of colorectal carcinoma in the Low_DC subgroup (at a dosage of 0.2 mg /kg of BW per day) and the High_DC subgroup (at a dosage of 2 mg /kg of BW per day) in comparison with the tumor control subgroup in a mouse xenograft model (*P*<0.05). By the end of the 8-week experimental period, both the Low_DC and High_DC subgroups showed a significant reduction in tumor weight (*P*<0.05) in comparison with the tumor control subgroup (Fig 6B). The histopathological staining results clearly indicated that treatment with DC inhibited tumor progression in these experimental animals (Fig 6C). No hepatotoxicity was found in the experimental subgroups, including the Low_DC and High_DC subgroups (Fig 6D). Our results showed that DC treatment significantly inhibited the growth of colorectal carcinoma in a mouse xenograft model.

## Discussion

CA is an effective agent against chronic inflammation. Previous studies suggested that CAPE, a derivative of CA, is an effective phytochemical for cancer prevention [20, 22, 23]. In the current study, we demonstrated that DC, a novel derivative of CA, significantly blocked the proliferation of human CRC cells *in vitro*. DC is a more effective agent against HT-29 cells (*KRAS* gene wild-type) than HCT-116 cells (*KRAS* gene mutant). Other studies suggested that an increased expression of cell-cycle regulators, such as cyclin A and cyclin E proteins, is associated with the excessive proliferation of human CRC cells [24]. In this study, our results demonstrated that DC effectively inhibited the proliferation of CRC cells through an increased expression of cell-cycle arrest at the S phase. The action of DC reduced the expression of cyclin A and cyclin E proteins in CRC cells including HCT-116 and HT-29 cells. It was recently reported that cyclin A and cyclin E are downstream target proteins of the PI3-K/Akt pathways in CRC cells. As illustrated in Fig 3, our results showed that DC was able to inhibit the PI3-K/Akt signaling pathways in human CRC cells. This suggests that the mechanism of action is in part through blocking the Akt signaling pathway in CRC HCT-116 and HT-29 cells. Interestingly, DC induced the formation of autophagy through the expression of autophagy proteins including Atg3, Atg16, Beclin-1 and LC3A/B in HCT-116 cells rather than in HT-29 cells. The results further indicated that DC induced an acquired resistance by triggering an autophagy reaction in HCT-116 cells rather than in HT-29 cells. Suppression of autophagy further enhanced cell death in DC-treated HCT-116 cells. These results provided an explanation of why DC is less effective in the suppression of HCT-116 cells than of HT-29 cells. Our results also confirmed that DC significantly inhibits the growth of colorectal carcinoma in a mouse xenograft model (Fig 6).

With the suppression of these critically important signaling pathways and regulatory proteins, DC can significantly inhibit the proliferation of these CRC cells (Fig 1). Indeed, our results showed that DC differentially inhibited several key proliferation pathways in CRC cells. DC could specifically target some of these important cell survival cascades. For example, DC specifically blocked the activation of the Akt signaling molecule in CRC cells. Due to the decreased expression of cell-cycle regulators such as cyclin A and E proteins, it is plausible that DC could effectively inhibit the proliferation of CRC cells through cell arrest at the S phase (Fig 2).

These findings are consistent with our previous reports and demonstrate the importance of DC as an effective inhibitor of the Akt and STAT3 signaling pathways. DC inhibited cell proliferation and induced cell-cycle arrest through the suppression of cell-cycle regulators such as cyclin A and cyclin E proteins in CRC cells. Unexpectedly, DC induced cell-protection action through an induction of autophagy in HCT-116 cells instead of in HT-29 cells due to the KRAS mutation background. The blockade of autophagy induced cell death and an increased sensitivity to DC in HCT-116 cells. A recent study indicated that treatment of the autophagy inhibitor would enhance cell death in CRC cells [25]. Our results were in line with the findings of previous studies [18, 25]. To date, this is the first evidence demonstrating the inhibitory effects of DC on the proliferation of human CRC cells both *in vitro* and *in vivo*. These results make DC a good candidate for future drug-design studies and for use as an anticancer therapeutic agent for CRC.

## Supporting information

S1 Fig DC induces cell-cycle arrest at the S phase through the suppression of cyclin A protein in CRC cells. (DOCX)

S2 Fig DC inhibited the cell proliferation of CRC cells through the inactivation of Akt protein. (DOCX)

S3 Fig Autophagy plays an important role in DC-mediated cell cytotoxicity in HCT-116 cells. (DOCX)

S1 Data The ARRIVE guidelines checklist. (PDF)

## Abbreviations

CAPE: caffeic acid phenethyl ester
CRC: Colorectal cancer
DC: decyl caffeic acid
EC: ethyl caffeic acid
mTOR: mammalian target of rapamycin
PI3-K: phosphatidylinositol-3-kinase
