# Supplementary material for: Decyl caffeic acid inhibits the proliferation of colorectal cancer cells in an autophagy-dependent manner in vitro and in vivo
Source: PLoS One. 2020 May 13;15(5):e0232832. doi: 10.1371/journal.pone.0232832 (PMC7219744; doi:10.1371/journal.pone.0232832)

**S1 Fig. DC induces cell-cycle arrest at the S phase through the suppression of cyclin A protein in CRC cells**

**Fig. 2C**

**HCT-116**

Cyclin A


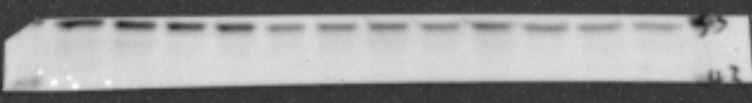


Cyclin E


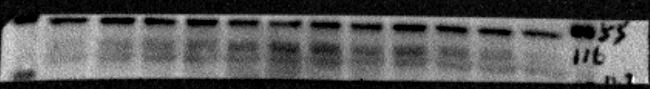


Cyclin B


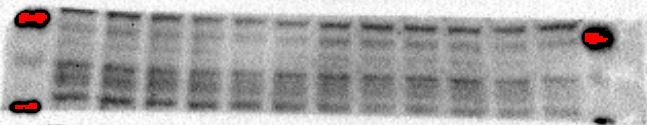


CDK2


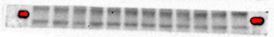


Lamin A


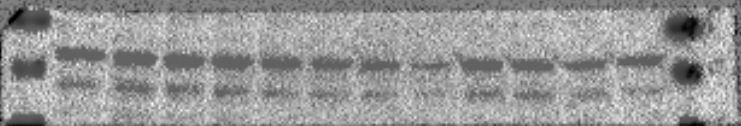


**HT-29**

Cyclin A


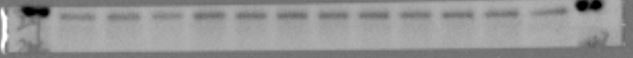


Cyclin E


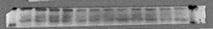


Cyclin B


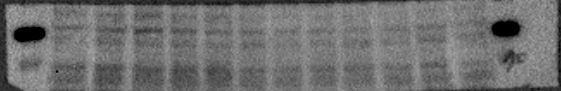


CDK2


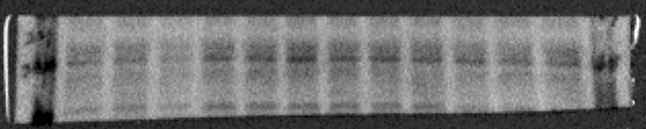


Lamin A


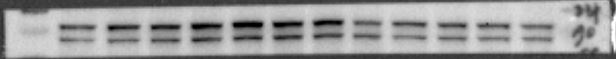

Supplement: S1 Fig — (DOCX) [file pone.0232832.s001.docx]
