# Supplementary material for: Decyl caffeic acid inhibits the proliferation of colorectal cancer cells in an autophagy-dependent manner in vitro and in vivo
Source: PLoS One. 2020 May 13;15(5):e0232832. doi: 10.1371/journal.pone.0232832 (PMC7219744; doi:10.1371/journal.pone.0232832)

**S2 Fig. DC inhibited the cell proliferation of CRC cells through the inactivation of Akt protein**

Fig. 3E

**HCT-116**

p-Akt


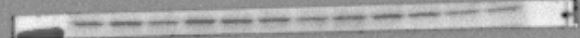


t-Akt


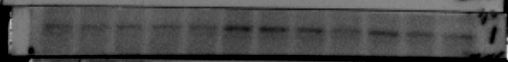


p-STAT3


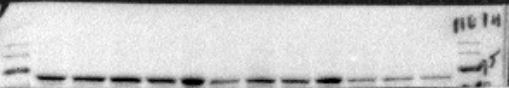


t-STAT3


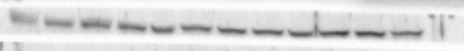


actin


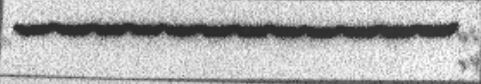


**HT-29**

p-Akt


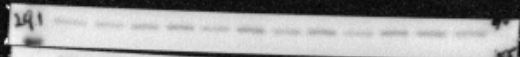


t-Akt


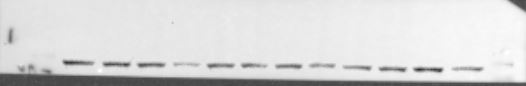


p-STAT3


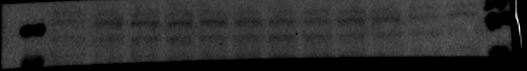


t-STAT3


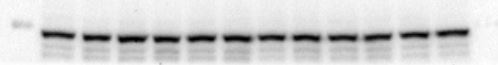


actin


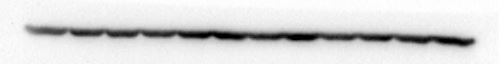

Supplement: S2 Fig — (DOCX) [file pone.0232832.s002.docx]
