# Supplementary material for: Decyl caffeic acid inhibits the proliferation of colorectal cancer cells in an autophagy-dependent manner in vitro and in vivo
Source: PLoS One. 2020 May 13;15(5):e0232832. doi: 10.1371/journal.pone.0232832 (PMC7219744; doi:10.1371/journal.pone.0232832)

**S3 Fig. Autophagy plays an important role in DC-mediated cell cytotoxicity in HCT-116 cells**

**Fig. 4E**

**HCT-116**

Atg3


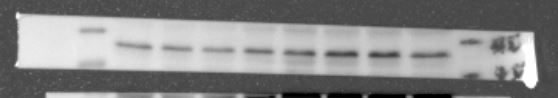


Atg 5


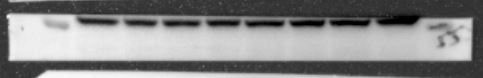


Atg16


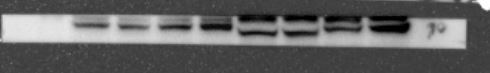


Atg12


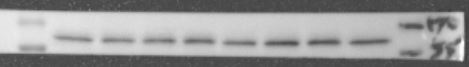


Beclin1


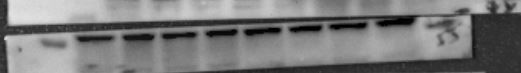


LC3A/B


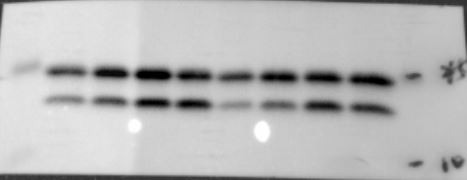


actin


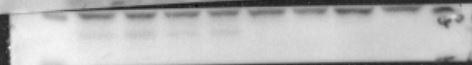


**HT-29**

Atg3


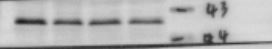


Atg 5


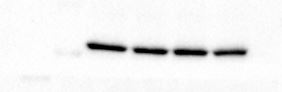


Atg 16


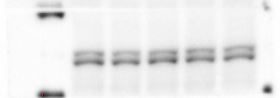


Atg 12


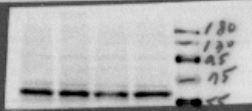


Beclin1


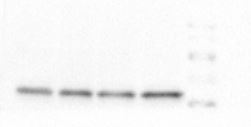


LC3A/B


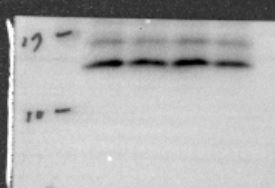


actin


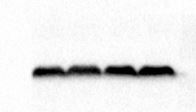


**Fig. 4G**

**HCT-116**

LC3A/B


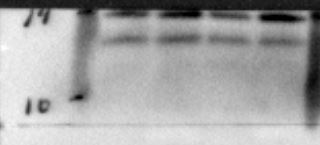


Actin


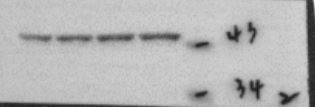


**HT-29**

LC3A/B


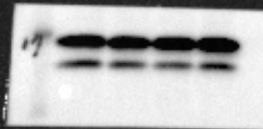


Actin


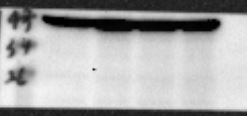

Supplement: S3 Fig — (DOCX) [file pone.0232832.s003.docx]
